# Predictive Factors for Successful Treatment of Deep Incisional Surgical Site Infections following Instrumented Spinal Surgeries: Retrospective Review of 1832 Cases

**DOI:** 10.3390/diagnostics12020551

**Published:** 2022-02-21

**Authors:** Masahiro Kuroiwa, Jordy Schol, Daisuke Sakai, Natsumi Horikita, Akihiko Hiyama, Hiroyuki Katoh, Yukihiro Yamamoto, Masato Sato, Masahiko Watanabe

**Affiliations:** 1 Department of Orthopaedic Surgery, Tokai University School of Medicine, Isehara 259-1193, Japan; 9618c4fish@gmail.com (M.K.); schol.jordy@gmail.com (J.S.); natsumi.horikita7@gmail.com (N.H.); a.hiyama@tokai-u.jp (A.H.); hero@tokai-u.jp (H.K.); yukihiro-tksr@tokai-u.jp (Y.Y.); sato-m@is.icc.u-tokai.ac.jp (M.S.); masahiko@is.icc.u-tokai.ac.jp (M.W.); 2 Department of Orthopaedic Surgery, Tokai University Hachioji Hospital, Tokyo 192-0032, Japan

**Keywords:** spine, surgery, instrumentation, surgical site infection, prognostic factor, antibiotics, postoperative infection, MRSA

## Abstract

Background: Surgical site infection (SSI) is a major complication in spinal instrumentation that is often difficult to treat. The purpose of this study was to identify and determine prognostic indicators for successful treatment of spine instrumentation SSI. Methods: Retrospectively, spine surgery cases were examined on SSI diagnosis. Post-instrumentation SSI patients were categorized as “Successful” if SSI subsided after single debridement. Patients in whom SSI did not subsided and/or required removal of instrumentation were classified as “Challenging”. We investigated the relation of treatment outcomes to patients and treatment factors. Results: A total of 1832 spinal instrumentation cases were recognized with 44 (2.40%) SSI cases. White blood cell count, C-reactive protein (CRP) levels, causative bacteria (i.e., *S. Aureus* or MRSA), trauma injury, and early-stage antimicrobial agent sensitivity correlated with treatment prognosis. Multivariate analysis highlighted CRP levels and applying early-stage sensitive antibiotics as potential impactful predictive factors for successful treatment. Conclusions: Our results demonstrated that early selection of sensitive antimicrobial agents is critical and emphasizes the potential for early-stage classification methods such as Gram staining. Additionally, *S. Aureus* and MRSA SSI formed significantly more challenging infections to treat, thus requiring consideration when deciding on instrumentation retention. These factors offer promising aspects for further large-scale studies.

## 1. Introduction

Surgical site infection (SSI) is a major complication of concern for spinal instrumentation. Spinal surgery SSI knows a relatively high incidence, of 1.9% to 6.3% [1,2,3,4], but remains relatively understudied [5]. Specifically, for spinal instrumentation, high SSI rates have been reported. For example, rates of 2.6% and 3.7% for instrumented spinal fusion have been reported [6,7], as well as rates of 11.9% in a myelomeningocele or cerebral palsy instrumented scoliosis fusion cohorts [8]. This consequently leads to higher morbidity rates, worsening long-term outcomes and increases in medical expenditure [9,10]. Multiple risk factors for SSI have been determined, e.g., diabetes, male sex, age of 60 years or older, smoking, previous surgical infection, increased body mass index, alcohol abuse, use of allograft, prolonged operation time, and prolonged duration of closed suction drainage [1,4,8,11,12,13,14]. Additionally, we previously determined that spine trauma injury and insufficient intraoperative irrigation are risk factors for spinal SSI [1]. Nonetheless, predictive factors for successful SSI resolution have not been well studied. Predictive aspects such as causative bacteria and diabetes have been suggested to be related to prognosis of post-spinal surgery SSI; however, these factors were found in a general cohort of spinal surgery patients, including cases without instrumentation [15,16]. Instrumentation surgery knows higher rates of SSI and overall shows enhanced rates of resistance to anti-microbial intervention [15,16]. Methicillin-resistant (MR) microbial pathogens have been presented as a negative prognostic factor for spine surgery SSI [15,16], including severe worsening of prognosis for spinal instrumentation SSI clearance [17].

Particularly due to biofilm formation, SSI occurrence after instrumentation surgery is implicated with a decision regarding removal or preservation of the implanted instruments. An optimal approach regarding instrument preservation with SSI remains controversial, where some advocate direct removal while others promote approaches to retain the instruments [18,19]. Removal of the implants can lead to deformity deterioration, promote mechanical instability, and limit initiated spinal fusion, and is linked with a general risks associated with invasive spinal surgeries, such as neurological damage. Despite the controversy, it is crucial to have predictive values that can be used to anticipate the success of SSI treatment, potentially reducing the need for instrumentation removal. Nonetheless, limited reporting is available to give a clear indication of which prognostic factors are associated with SSI resolution. Thus, to enhance comprehension on desirable management of instrumentation SSI, we retrospectively examined cases of spinal surgery at our medical institution comparing successfully treated cases of instrumentation SSI, i.e., single debridement with preservation of the implants leading to SSI clearance compared to instrumentation SSI cases requiring multiple interventions and/or implant removal, in order to identify prognostic factors that correlated with successful treatment.

## 2. Materials and Methods

**Figure 1:**
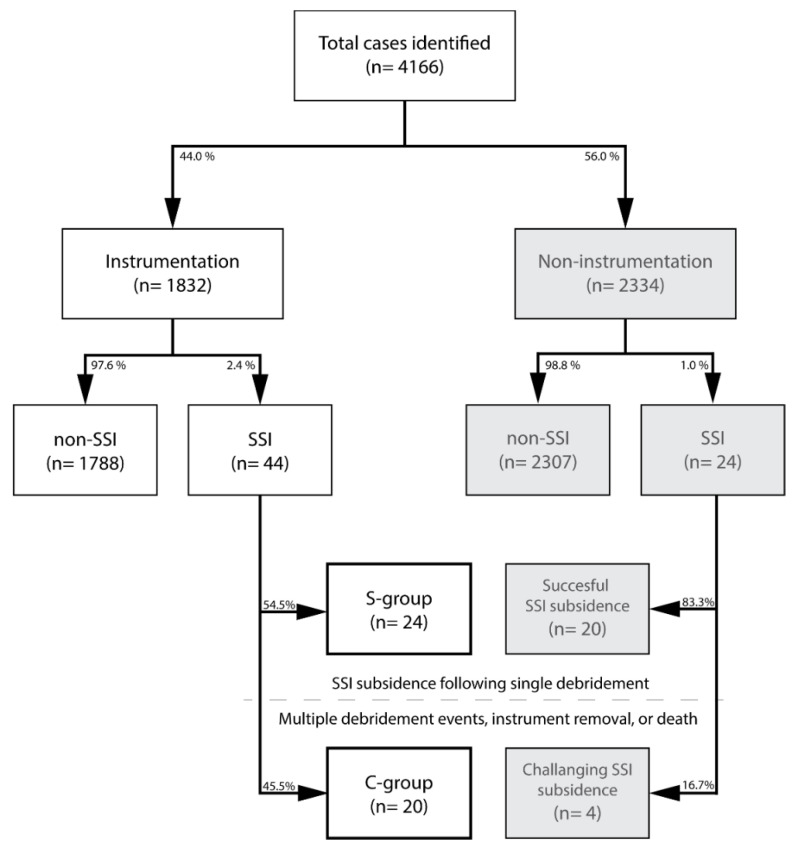
Flow chart depicting the study population and their respective categorization. Abbreviations: SSI, surgical site infection occurring post-spinal surgery; S-group, successful treatment of SSI with single debridement in the instrumentation cohort; C-group, instrumentation cohort with SSI that underwent multiple debridement interventions, instrumentation removal, or succumbed to death.

All procedures described here were performed upon approval of our institutional review board. Informed consent for analysis of medical records was provided by participants or from their respective parent or legal guardian concerning subjects under the age of 18 years. We retrospectively reviewed all medical patient notes at the Tokai University School of Medicine Hospital department of Orthopaedic Surgery for spinal surgery cases treated between 2005 to 2015. All spine surgery cases were thereafter qualified into either instrumented surgery, which involved surgical intervention applying metallic implants such as pedicle screws, hooks, rods, plates, or cages, and non-instrumented spine surgery cases. These cohorts were further qualified based on the presence and diagnosis of deep incisional SSI post-spinal surgery (Figure 1). Deep incisional SSI was diagnosed in accordance with the definition set out by the Centers for Disease Control and Prevention guidelines [20]. Cases involving infections other than deep incisional SSI or nonfatal SSI cases for which the postoperative follow-up period was less than 1 year were excluded. All cases of SSI were examined for the causative microbial agent through two consecutive standard Gram-positive and -negative cultures. Prior to identification of the causative organism, first-generation cephalosporin antibiotic agents were intravenously administered as a dose per patient body weight [21]. Following causative microbial identification, sensitive antibiotics were applied intravenously for at least 6 weeks.

Identified cases of post-instrumentation deep incisional SSI were further categorized into two separate cohorts. The first cohort involved SSI patients who showed successful subsidence of the infection by single debridement intervention together with an antibiotic course and did not require removal of implanted instruments (S-group). Successful SSI subsidence was qualified as negative microbial cultures and normalized blood values 6 weeks following post-diagnosis and antibiotic treatment. The second cohort included challenging cases (C-group) in which the SSI did not subside after the initial debridement, and thus were subjected to two or more debridement interventions. Additionally, patients for whom instrumentation was removed or SSI unfortunately led to death were categorized into the C-group. From both cohorts, preoperative factors including age, sex, medical history related to risk factors for SSI (i.e., diabetes, rheumatoid arthritis, and hemodialysis), and spinal injury trauma were examined. Similarly, postoperative factors were investigated, which included the number of fixated vertebral bodies, time of SSI diagnosis, white blood cell count upon onset, C-reactive protein (CRP) levels upon onset, methicillin-resistant *Staphylococcus aureus* (MRSA) involvement, and the presence of bacteremia. Aspects of primary SSI treatment regime, i.e., sensitivity of initially antimicrobial agents administered (5–7 days after diagnosis until exudate culture results were determined), number of days from diagnosis until debridement, operation time during debridement, volume of blood loss, volume of intraoperative saline irrigation, application of continuous closed irrigation, vancomycin powder dispersion over the operative field, and duration of postoperative closed suction drain placement, were also examined.

The data in this study are displayed as mean ± standard deviation. Statistical analysis was performed through Prism 9 for MacOS (version 9.3.0, GraphPad Software LLC, San Diego, CA, USA). Data normality was assessed through the Shapiro–Wilk test. Groups were compared through the Mann–Whitney U test for non-parametric data and Unpaired *t*-Test for parametric values. Categorical variables were analyzed through Fisher’s exact test. Finally, a multinomial logistic regression analysis was performed through IBM^®^ SPSS^®^ statistics version 26 (IBM SPSS, Foster City, CA, USA) to test for correlations between factors and treatment prognosis. The level of statistical significance was set below 5%.

## 3. Results

### 3.1. Patient Selection

**Figure 2:**
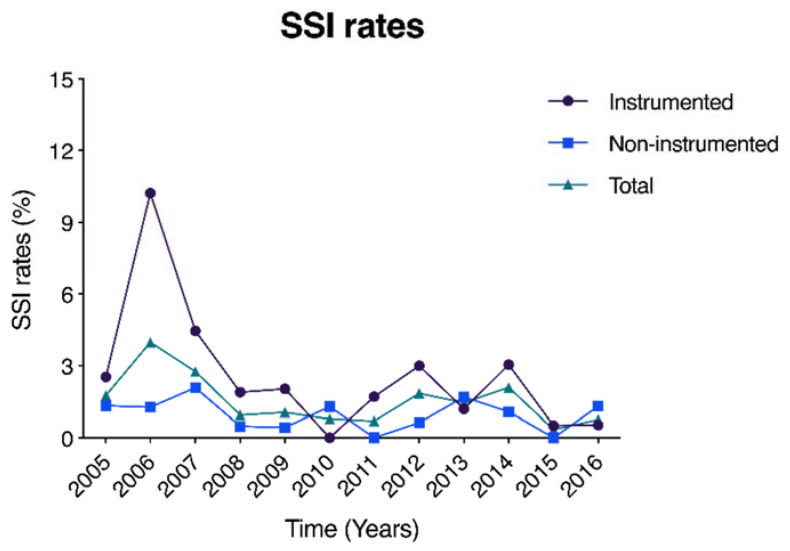
Graphical representation of deep surgical site infection (SSI) rates over time, given as percentage of all instrumented and non-instrumented surgical spine cases.

From a register of 4166 cases of spinal surgery, we identified 1832 cases of spinal instrumentation (Figure 1). Deep incisional SSI was diagnosed in 68 of 4166 spinal surgery cases. The SSI incidence showed a gradual decreasing trend over time from 3.98% in 2006 to 0.75% in 2015 (Figure 2). Overall, SSI post-instrumentation surgery counted 44 of 1832 cases (2.40%) compared to 24 of 2334 cases (1.03%) of spinal surgery without instrumentation.

### 3.2. Surgical Site Infection Characteristics

The time of SSI diagnoses in the total cohort was 18.6 ± 11.0 days postoperatively. The primary signs for SSI diagnosis were pus discharge from the wound or dehiscence of the wound. The most common causative bacterium was determined as *S. aureus* in 32 of 68 cases (47.1%), followed by coagulase-negative *staphylococcus* (CoNS) in 25 (36.8%) patients. The causative bacterium remained unknown due to negative cultures in nine records (13.2%) (Table 1). Moreover, MR was recorded in 44 of 68 patient cultures (79.4%) involving 24 cases of *S. aureus* and 18 cases involving CoNS. When SSI occurred after spinal instrumentation, *S. aureus* was the most common causative bacterium (21 of 44, at 47.7%), of which 16 (76.2%) showed MR. No higher rates of MR were observed in the instrumented versus non-instrumented group (*p* = 0.288) Of note, however, was the lack of Gram-negative SSI in the non-instrumented cohort.

### 3.3. Instrumentation SSI Subsidence

Of the 44 cases of instrumentation SSI, 24 (54.5%) were included in the S-group and 20 (45.5%) in the C-group, which showed a significantly (*p* = 0.020) higher treatment failure rate than the non-instrumentation, successfully treated SSI cohort (4 of 24 (16.7%)) (Figure 1). There was no significant difference between S-group and C-group regarding age, sex, medical history, number of fixated vertebrae, or number of postoperative days until SSI onset (Table 2). A significantly (*p* = 0.006) higher number of spinal trauma injury cases were found in the C-group (70.0%) than in the S-group (25.0%). Values for white blood cell count (*p* = 0.002), CRP (*p* = 0.001), and the frequency of MRSA as the causative bacterium (60.0% vs. 16.7%, *p* = 0.005) were significantly higher in the C-group compared with those in the S-group (Table 2).

Antimicrobial agents were administered upon diagnosis of SSI. Retrospectively, application of a sensitive agent to the causative bacteria during the initial period was achieved in 88.9% of the cases in the S-group, while only in 45.0% of the cases in the C-group, demonstrating a significantly (*p* = 0.006) lower rate of correct antibiotic regime in C-group. No significant differences were observed between the two groups with respect to any of the other treatment factors.

### 3.4. Multivariate Analysis

Subsequent multinomial logistic regression on the six variables of spinal trauma, white blood cell count, CRP levels, MRSA or *S. Aureus* as causative bacteria, and antimicrobial agent sensitivity was performed. The results of multivariate analysis using these six factors as explanatory variables revealed no significant odd ratios, although CRP levels with an odds ratio of 1.11 and antibiotic sensitivity with an odds ratio of 0.08 approached statistical significance with a *p* value of 0.054 and 0.082, respectively (Table 3).

## 4. Discussion

### 4.1. SSI Characteristics

Our study revealed an SSI rate of 1.6% of all included cases, which is in line with a comprehensive systematic review of Patel et al., concluding a pooled 1.9% SSI rate for general spine surgery [22]. Spine instrumentation rates were slightly higher in our report (i.e., 2.4%), which was markedly lower than the pooled 3.8% rate from the beforementioned review [22]. Previous reporting identified *S. aureus*, followed by CoNS, which includes *S. epidermis,* as the primary post-spinal surgery SSI causative microbes [2,3,23,24]. Our study similarly suggested *S. aureus* (47.7%) and CoNS (31.8%) as the primary causes of SSI post-instrumentation spinal surgery. Further, we reported a relatively high rate of MR bacteria in overall SSI cases (64.7%). Particularly, within the spinal trauma cohort, high rates of MRSA (65% of instrumented trauma patients) were examined, and this cohort accounted for 81.3% of all MRSA cases among the instrumented trauma cases. Risk factors for MRSA infection were previously reported to include long hospitalization, intensive care unit (ICU) hospitalization, and a history of antimicrobial agents’ usage [25,26,27]. As most cases of spinal injury involve high-energy trauma, such cases often require extended hospitalization in the ICU, including long preoperative hospitalization and preoperative use of antimicrobial agents. This likely explains the high MR infection rate in cases of spinal trauma SSI. The incidence of MR is associated with increases therapeutic costs, morbidity, and mortality [17,28]. Multiple initiatives have been established since to combat the risk of MR SSI infections. At our institution, comprehensive pre-surgery showering and washing procedures have been initiated. Similar protocols and other screening procedures are being tested or adopted at other institutions to reduce the rate of MR SSI [29,30,31]. Although SSI rates overall are in decline, including at our institution (Figure 2), SSIs still occur, making countermeasures, to be taken upon occurrence, as important as prevention. On the contrary, overall rates of MR were relatively high (64.7% of patients) including the instrumented and non-instrumented surgery cases. Careful consideration should be given to the application of prophylactic antibiotics to minimize the spread of antibiotic-resistant species, in particular in view of the high rates of MRSA and other antibiotic-resistant species reported in Japan [32].

### 4.2. Prognostic Factors for Successful SSI Treatment

Multiple studies have aimed to identify risk factors related to treatment of spinal SSI. Work from 2014 by Maruo and Berven examined SSI following spinal surgery for both instrumentation and non-instrumentation surgery cases, and found late diagnosis of SSI (>90 days post-surgery) to be correlated with SSI treatment failure, while infection characteristics of MSSA and superficial SSI correlated with successful SSI subsidence [15]. Their study further suggested an approximately three times higher rate of SSI treatment failure in the instrumentation cohort compared to the non-instrumentation group. Dipaola et al. reported that patients requiring multiple debridements and irrigation of post-spinal surgery SSI are related to anatomical location, comorbidities, causative bacteria, distant site infection, and type of bone graft employed [16]. Moreover, their work further emphasized the application of instrumentation as a negative predictive factor for SSI treatment resolution. Nevertheless, while both studies underline the additional challenge for spinal instrumentation SSI resolution, they did not specifically examine predictive factors for successful treatment in these cohorts. Our study similarly found higher rates of SSI and subsequent significantly (*p* = 0.020) lower rates of successful SSI treatment within the instrumentation cohort. Of instrumentation SSI cases, 14 (31.8%) ultimately required instrument removal. Moreover, one patient died of sepsis associated with SSI. These findings further emphasize the difficulty of SSI treatment in cases of spinal instrumentation. The challenge toward successful SSI treatment for instrumented patients might partly be ascribed to the risk of biofilm formation of the causative bacteria on the metallic implants [33,34]. This biofilm environment has been shown to protect the microbes, increasing resistance to antibiotic agents as well as to cellular and humoral immune responses [34].

To the best of our knowledge, our study is the first to determine predictive factors for successful SSI treatment in spinal instrumentation cohorts. Our univariance analysis revealed multiple predictive factors, i.e., WBC count, CRP levels, MRSA, *S. Aureus*, antibiotic sensitivity, and trauma. A general look at our identified factors suggests two separate categories of predictive factors, i.e., the intensity of infection (as indicated by WBC and CRP levels) and the type/resistance of the bacteria, thus accentuating a strong advantage for SSI treatment by early SSI detection and specifically targeted anti-microbial agents. These findings are in line with previous reporting on general spinal SSI treatment, similarly suggesting CRP, antibiotic sensitivity, and causative bacteria as prognostic factors [15,17]. The initial period of antimicrobial agents in our study referred to the 5–7 days after SSI diagnosis until the exudate culture results were determined. The administration of antimicrobial agents was initiated after SSI diagnosis in all cases. Culture testing of exudate or infected tissue was also performed. Naturally, in all cases, the antimicrobial agent that exhibited sensitivity to the causative bacteria according to the cultures was administered after the culture results were determined (excluding the nine cases in which culture results were negative). Although the regimen was changed to an appropriate antimicrobial agent after the culture results were determined, application of non-sensitive antimicrobial drugs in this crucial, initial time point was determined to be an adverse prognostic factor for successful treatment of post-spinal instrumentation deep incisional SSI. This might be considered an obvious observation; nonetheless, it highlights the importance of speed in determining the pathogen. Although culture testing is a fundamental and extremely important element of SSI treatment, this procedure takes multiple days or weeks. Because the selection of the antimicrobial agent to be administered after SSI diagnosis until the culture results are determined can affect treatment prognosis, the causative bacteria must be carefully predicted and an appropriate antimicrobial agent must be selected in this pivotal stage of SSI treatment. Here, an antimicrobial agent with no sensitivity to the causative agent was initially administered in 13 instrumentation cases, including eight in which a non-MRSA agent was administered for a MRSA infection. Although agents to treat MRSA should be generally considered as the drug for initial administration, it is dangerous to select such agents blindly because four of 13 cases were administered anti-MRSA agents to treat Gram-negative bacilli of *Enterobacter cloacae* and *Pseudomonas aeruginosa*. As microbiology culture tests often take multiple days, it might prove beneficial to supplement culture testing with direct microscopic and Gram staining examination at the time of diagnosis. As such, procedures can be performed in approximately 30 min to allow for better prediction of the causative bacteria and consequently selecting a sensitive antibiotic agent within this crucial time frame. Additionally, new tools, techniques, and systems ideally will be developed to increase the success rates of SSI treatment, by increasing the likelihood of appropriate antibiotics’ selection. Moreover, our findings emphasize the vital importance of recording and increasing awareness of the frequency of appearance of each causative bacterium at the individual-facility level. It currently appears safe to first perform Gram staining of the exudate and/or infected tissue on the day of SSI diagnosis and then select an anti-MRSA agent if the results indicate Gram-positive cocci or antimicrobial agents, such as cefepime or meropenem, with high sensitivity against Gram-negative bacilli if a Gram-negative bacillus is identified. However, further research is needed to support and confirm this hypothesis.

### 4.3. Limitations

Because this was a retrospective study and SSI diagnosis was not definitive in two cases, the data for these cases were excluded. Some treatments, such as dispersion of vancomycin powder over the operative field and continuous closed irrigation, were performed in only a small number of cases. There also may have been other confounding factors, such as patient nutritional state and removal of bone graft during debridement. Data on more cases must be gathered so that this topic can be studied continuously going forward. Despite these limitations, we were able to identify some critical prognostic factors that should be carefully considered during the management of instrumented SSI in spinal surgeries.

## 5. Conclusions

In conclusion, our retrospective study found an overall incidence of spinal instrumentation SSI of 2.4%, compared to 1.0% for non-instrumentation cases. Moreover, SSI post-instrumentation was associated with higher numbers of SSI treatment failure. The success of treatment of spinal instrumentation deep incisional SSI was highly correlated with the application of a susceptible antimicrobial agent at the time of SSI diagnosis as well as CRP levels, WBC count, trauma injury, MRSA, and *S. Aureus* SSI. Our findings emphasize the importance of timely identification of the causative bacteria and highlight the need for awareness of the type of SSI bacteria frequencies at each institution and need for advancements in techniques supporting the prediction of causative bacteria at the early stage of SSI onset and diagnosis.

## Figures and Tables

**Table 1:** Culture results determining the bacterium causing post-spinal surgery deep incisional SSI, comparing between instrumented and non-instrumented spinal surgeries and the S-group to the C-group. The (%) represents the percentage of patients scoring positive for a particular microbe. MR: Methicillin-resistant.

			Total (n = 68)		No Instrumentation (n = 24)		Instrumentation (n = 44)		S-Group (n = 24)		C-Group (n = 20)	
	Family	Micro-Organism	n	(%)	n	(%)	n	(%)	n	(%)	n	(%)
Gram-negative	*Corynebacteriaceae*	Corynebacterium	1	1%	0	0%	1	2%	0	0%	1	5%
	*Enterobacteriaceae*	*Proteus mirabilis*	1	1%	0	0%	1	2%	0	0%	1	5%
		*Enterobacter cloaca*	1	1%	0	0%	1	2%	1	4%	0	0%
		*Escherichia coli*	1	1%	0	0%	1	2%	0	0%	1	5%
	*Moraxellaceae*	*Acinetobacter baumannii*	1	1%	0	0%	1	2%	0	0%	1	5%
Gram-positive	*Enterococcaceae*	*Enterococcus faecalis*	1	1%	0	0%	1	2%	1	4%	0	0%
	*Bacillaceae*	*Bacillus cereus*	1	1%	0	0%	1	2%	0	0%	1	5%
	*Staphylococcaceae*	*Staphylococcus capitis*	0	0%	0	0%	0	0%	0	0%	0	0%
		*Staphylococcus capitis-*MR	7	10%	3	13%	4	9%	4	17%	0	0%
		*Staphylococcus epidermidis*	3	4%	1	4%	2	5%	2	8%	0	0%
		*Staphylococcus epidermidis-*MR	11	16%	5	21%	6	14%	5	21%	1	5%
		*Staphylococcus schleiferi*	1	1%	1	4%	0	0%	0	0%	0	0%
		*Staphylococcus lugdunensis*	1	1%	0	0%	1	2%	0	0%	1	5%
		*Staphylococcus aureus*	6	9%	1	4%	5	11%	3	13%	2	10%
		*Staphylococcus aureus-*MR	26	38%	10	42%	16	36%	4	17%	12	60%
Other		*Unknown*	9	13%	3	13%	6	14%	6	25%	0	0%
		Cases with polymicrobial infection	3	4%	0	0%	3	7%	2	8%	1	5%
		Methicillin-resistant species	44	64.7%	18	75%	26	59%	14	58%	12	60%

**Table 2:** Demographic and treatment characteristics for successful, instrumented, deep surgical site infection treatment by single debridement (S-group) compared to unsuccessful treatment, thus requiring multiple debridement interventions and/or removal of the implanted instruments or resulted in death (C-Group). A; selection of sensitive antibiotic treatment prior to microbe examination. Assessed through * Mann–Whitney U test, ** Unpaired *t*-test, or *** Fisher’s exact test. A: presence of diabetes, rheumatoid arthritis, or hemodialysis as risk factors for SSI; B: selection of effective antibiotic regimen against SSI causative bacteria at initial week of onset. Abbreviations: CoNS, coagulase-negative staphylococcus; CRP, C-reactive protein; MRSA, Methicillin–resistant *Staphylococcus aureus*; SSI, surgical site infection; sd, standard deviation; and WBC, white blood cells. Statistical significant *p*-values are marked in bold.

		Total			S-Group			C-Group			
Factor	(unit)	Mean	±sd	n	Mean	(±sd)	n	Mean	(±sd)	n	*p*-Value
Age	(years)	50.7	±20.1	44	20.0	±20.0	24	56.2	±19.4	20	0.056 *
Fixated vertebrae	(vertebrae)	4.7	±3.4	44	5.3	±4.0	24	4.0	±2.3	20	0.642 *
Time onset SSI	(days post-surgery)	34.7	±107.1	44	15.9	±8.5	24	57.3	±157.7	20	0.986 *
WBC count	(WBC/μL)	10,034.1	±3964.5	44	8370.8	±2007.7	24	12,030.0	±4796.3	20	0.002 **
CRP levels	(mg/dL)	10.2	±9.3	44	6.4	±8.0	24	14.8	±8.9	20	0.001 *
Time until debridement	(days)	2.4	±2.7	44	2.2	±2.8	24	2.6	±2.5	20	0.388 *
Debridement time	(min)	99.8	±38.2	43	97.8	±34.5	24	102.4	±43.1	19	0.695 **
Blood loss	(g)	352.2	±329.5	40	388.4	±365.0	22	308.0	±284.1	18	0.545 *
Volume saline irrigation	(mL)	14,848.8	±8686.7	43	13,541.7	±5633.6	24	16,500.0	±11,417.6	19	0.649 *
Time of closed suction drain	(days)	5.9	±2.6	33	5.6	±2.2	21	6.6	±3.1	12	0.363 *
		**n**	**%**	**N**	**n**	**%**	**N**	**n**	**%**	**N**	***p*** -value ***
Sex	(male: female)	18:26		44	12:12			6:14			0.227
Medical history ^A^		15	34.9%	43	9	37.5%	24	6	31.6%	19	0.755
Trauma		20	45.5%	44	6	25.0%	24	14	70.0%	20	**0.006**
Continious closed irrigation		3	6.8%	44	1	4.2%	24	2	10.0%	20	0.583
Bacteremia		25	56.8%	44	13	54.2%	24	12	60.0%	20	0.766
Vancomycin powder		7	15.9%	44	4	16.7%	24	3	15.0%	20	>0.999
Antibiotic sensitivity ^B^		25	65.8%	38	16	88.9%	18	9	45.0%	20	**0.006**
Gram-positive species		34	77.3%	44	17	70.8%	24	17	85.0%	20	0.306
CoNS		14	31.8%	44	11	45.8%	24	3	15.0%	20	0.050
*S. Aureus*		21	47.7%	44	7	29.2%	24	14	70.0%	20	**0.014**
Gram-negative species		5	11.4%	44	1	4.2%	24	4	20.0%	20	0.161
MRSA frequency		16	36.4%	44	4	16.7%	24	12	60.0%	20	**0.005**

**Table 3:** Risk factor assessment for successful treatment of post-instrumentation SSI debridement, via multinomial logistic regression analysis, comparing the C-group to the reference S-group. Abbreviations: OR, odds ratio; CI, confidence interval; CRP, C-reactive protein; MRSA, methicillin-resistant *Staphylococcus aureus*; and WBC, white blood cells.

Prognostic Factor	OR	95% CI	*p* Value
WBC count	1.00	(1.00–1.00)	0.134
CRP levels	1.11	(1.00–1.24)	0.054
Trauma	1.26	(0.12–12.86)	0.845
Antibiotic sensitivity	0.08	(0.01–1.30)	0.082
*S. Aureus*	0.56	(0.02–15.23)	0.728
MRSA	14.52	(0.26–798.52)	0.191

## Data Availability

Data can be requested from the corresponding authors upon reasonable request.
